# Renal cortical siderosis: imaging findings and clinical relevance

**DOI:** 10.1093/bjrcr/uaag043

**Published:** 2026-10-15

**Authors:** Amar S Shah, Kyle J Chen, Mark Sugi

**Affiliations:** Department of Radiology, Mayo Clinic in Arizona, Phoenix, AZ 85054, United States; Department of Radiology, Mayo Clinic in Arizona, Phoenix, AZ 85054, United States; Department of Radiology, Mayo Clinic in Arizona, Phoenix, AZ 85054, United States

**Keywords:** renal cortical siderosis, hemolysis, atypical hemolytic uremic syndrome, MRI, kidney injury

## Abstract

Renal cortical hemosiderosis is the accumulation of iron in the renal cortex. On MRI, this can be inferred by marked cortical T2 hypointensity and loss of signal on in-phase imaging relative to opposed-phase imaging. Although classically associated with paroxysmal nocturnal hemoglobinuria, renal hemosiderosis can be seen in a variety of pathologic conditions associated with acute or chronic renal injury. We present a case of a 59-year-old female that developed acute kidney injury while hospitalized for management of hydronephrosis and hepatic abscess with imaging features on MR angiography and renal parenchymal biopsy consistent with renal cortical hemosiderosis. Combined with other laboratory results, this finding supports the diagnosis of atypical hemolytic uremic syndrome, a condition with poor prognosis for which timely treatment is essential. Thus, recognition of renal cortical hemosiderosis on imaging may facilitate appropriate clinical workup and timely management.

## Introduction

Renal cortical hemosiderosis, corresponding to hemosiderin deposition in the renal cortex, is an uncommon but important imaging finding that may be seen in patients with acute or chronic renal injury and is highly specific for intravascular hemolysis. MRI can be used to identify renal cortical hemosiderosis due to the paramagnetic effects of hemosiderin. Although renal hemosiderosis in and of itself is not the cause of renal injury, its identification on MRI can prompt further clinical investigation into the underlying cause and guide appropriate treatment. Common causes of renal cortical hemosiderosis include paroxysmal nocturnal hemoglobinuria (PNH), sickle cell anemia, and mechanical hemolysis, most commonly from mechanical cardiac valves.^1^

Intravascular hemolysis is characterized by anemia, low haptoglobin, elevated lactate dehydrogenase, as well as schistocytes. Typically, free hemoglobin binds to haptoglobin and is subsequently metabolized by the liver. However, in intravascular hemolysis, haptoglobin is depleted and free hemoglobin remains in circulation. It is then filtered by the kidney and either excreted in the urine or reabsorbed into the renal cortex and stored as hemosiderin.^2^ This may be recognized as a relative loss of T2 signal intensity and loss of signal intensity on in-phase imaging (compared to opposed-phase) on MRI.

We present the case of a 59-year-old female who developed acute renal injury in the setting of atypical hemolytic uremic syndrome (aHUS), diagnosed following renal biopsy demonstrating thrombotic microangiopathy (TMA) with subsequent confirmatory serum testing. In retrospect, renal cortical siderosis had developed contemporaneously with the patient’s declining renal function, and its recognition may have facilitated earlier diagnosis without the need for biopsy.

## Clinical presentation

A 59-year-old female with a history of systemic lupus erythematosus (SLE, diagnosed in the setting of thrombocytopenia, treated with platelet transfusion in a previous hospitalization, and anemia without blood transfusions), cryptogenic cirrhosis with cholestasis managed with biliary stenting, and cervical cancer treated with brachytherapy was hospitalized for management of right-sided hydroureteronephrosis (relieved with stenting) and hepatic abscesses, during which time she developed acute kidney injury (AKI), which persisted for several weeks. The patient received antibiotics—which consisted of metronidazole, a single dose of vancomycin, piperacillin-tazobactam, fluconazole, micafungin, meropenem, daptomycin, and ceftriaxone over the course of a month—and percutaneous drainage for the hepatic abscesses. Of note, the patient received a dose of vancomycin and 11 days of piperacillin-tazobactam, both of which are known to be nephrotoxic. The patient also received 3 units of packed red blood cells and tested negative for anti-phospholipid antibodies during this hospitalization.

The patient’s serum creatinine rapidly increased over a span of 2 weeks from 1.4 to 6.9 mg/dL despite maintenance of adequate urine output. Given her complicated medical history, the renal injury was thought to be multifactorial, related to systemic antibiotics, lupus, and infection. Urinalysis was notable for macroscopic hematuria and proteinuria.

## Differential diagnosis

The differential diagnosis for AKI is broad. Given this patient’s complex medical history, multiple causes of AKI were considered, including lupus nephritis and associated infarct, obstructive uropathy, prerenal insult, hepatorenal disease, and medication-related nephrotoxicity.

## Investigation/imaging findings

An MR angiogram was ordered to evaluate for renal infarct given the patient’s history of SLE, 2 months after the onset of AKI (time point = x + 2 months). MR angiography was notable for marked T2 hypointensity of the renal cortex (Figure 1A) relative to the medulla. On in-phase imaging, there was loss of signal intensity along the renal cortices. On out-of-phase imaging, a subtle type 2 chemical shift artifact (India ink artifact) outlined the renal cortices. Imaging was also notable for marked T2 hypointensity of the liver and spleen with accompanying relative loss of signal on in-phase imaging consistent with iron deposition (Figure 2). The pancreas and heart were of normal signal intensity, excluding primary causes of hemosiderosis (primary hemochromatosis). There was no altered perfusion or infarct in the kidneys, and the hydronephrosis had been managed with stenting.

**Figure 1:**
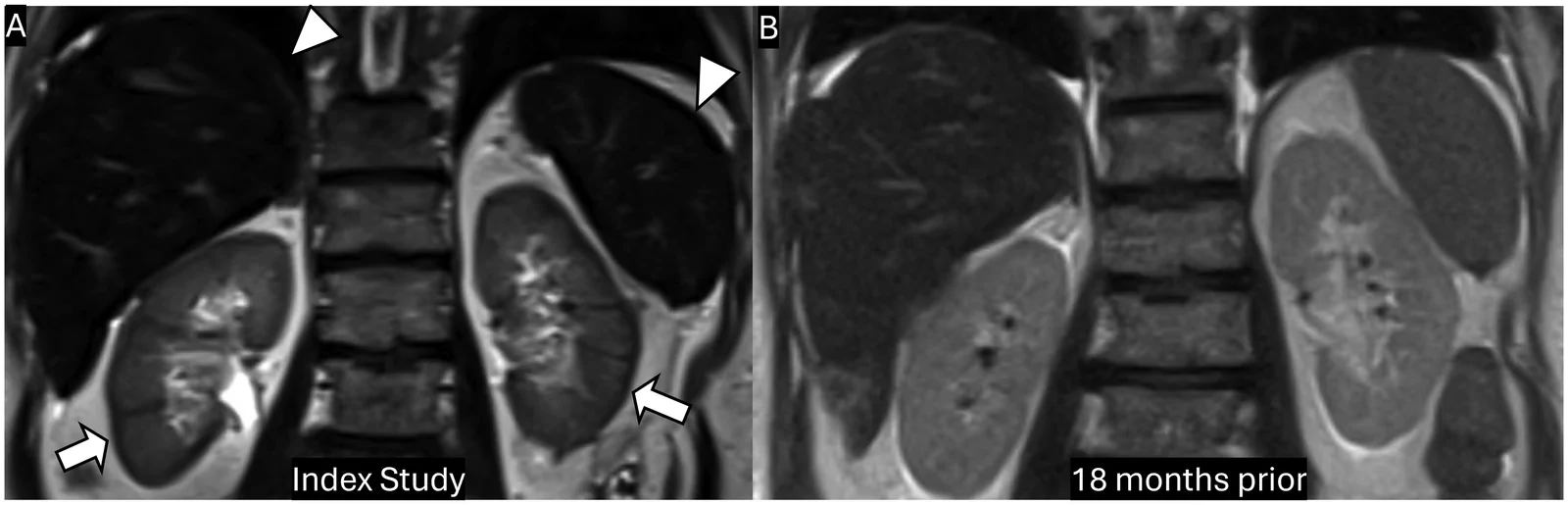
59-year-old female with cirrhosis and acute kidney injury (AKI) undergoing imaging for possible renal infarct (index study 2 months following AKI onset, X + 2). (A) Representative coronal T2w image demonstrates a rim of T2 hypointensity along the renal cortices (arrows), particularly compared to a study obtained 18 months prior (B). Hepatic and splenic T2 hypointensity is also seen, compatible with secondary siderosis (arrowheads).

**Figure 2:**
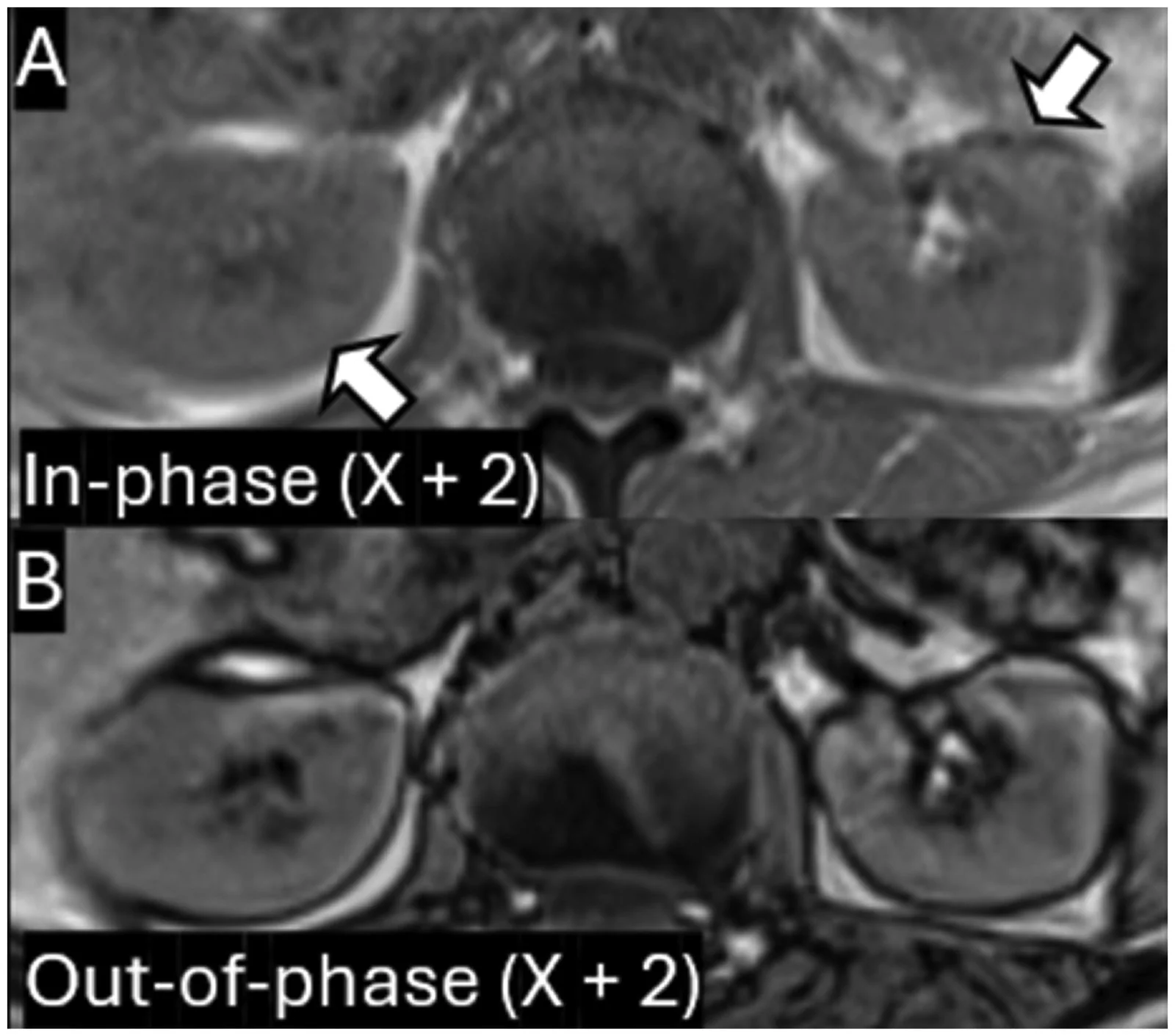
Axial T1w in-phase (A, top) and out-of-phase (B, bottom) images demonstrate subtle loss of signal intensity on in-phase acquisitions (arrows). Note the chemical shift artifact on out-of-phase imaging along the periphery of the kidneys obfuscating the underlying preserved signal intensity.

In retrospect, comparison with prior studies obtained around the time the patient had developed AKI (time point x, Figure 1B) and several months earlier on a hepatocellular carcinoma screening study (Figure 1C) demonstrated progressive loss of T2 signal in the renal cortices, more prominent after 2 months even allowing for some degree of technical differences (Figure 3). A similar time course can be seen with the evolution of systemic siderosis (Figure 4).

**Figure 3:**
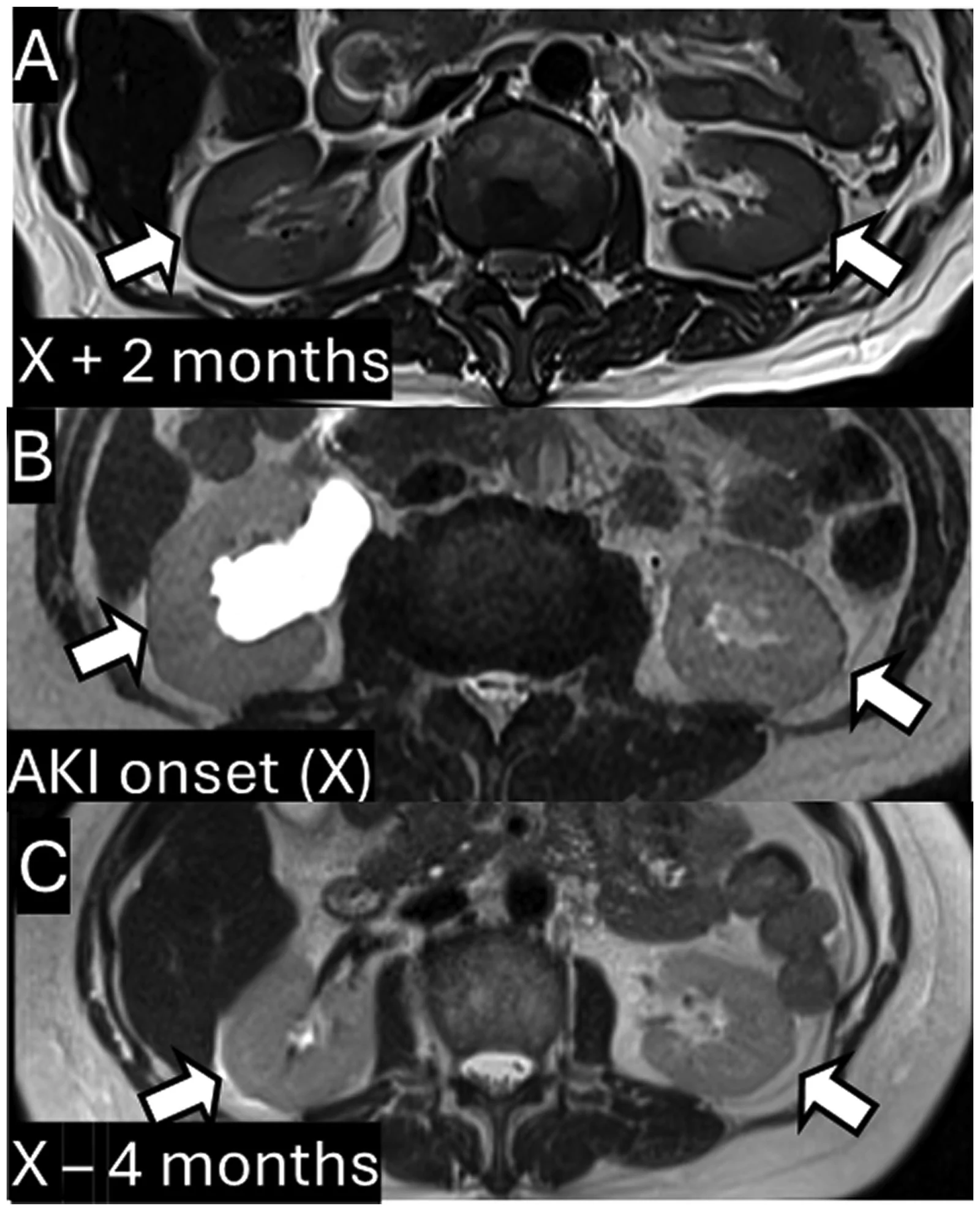
59-year-old female with cirrhosis and acute kidney injury (AKI) undergoing imaging for possible renal infarct. (A) Representative axial T2-weighted images from the index (time point 2 months following AKI onset, X + 2) study show a rim of low signal around the renal cortex bilaterally (arrows). (B) MRI obtained 2 months prior, at the time of onset of AKI (time point X) shows subtle cortical T2 hypointensity, best appreciated when compared to an earlier study 4 months prior to onset of AKI (time point X – 4, C), which shows a normal appearance of the kidneys.

**Figure 4:**
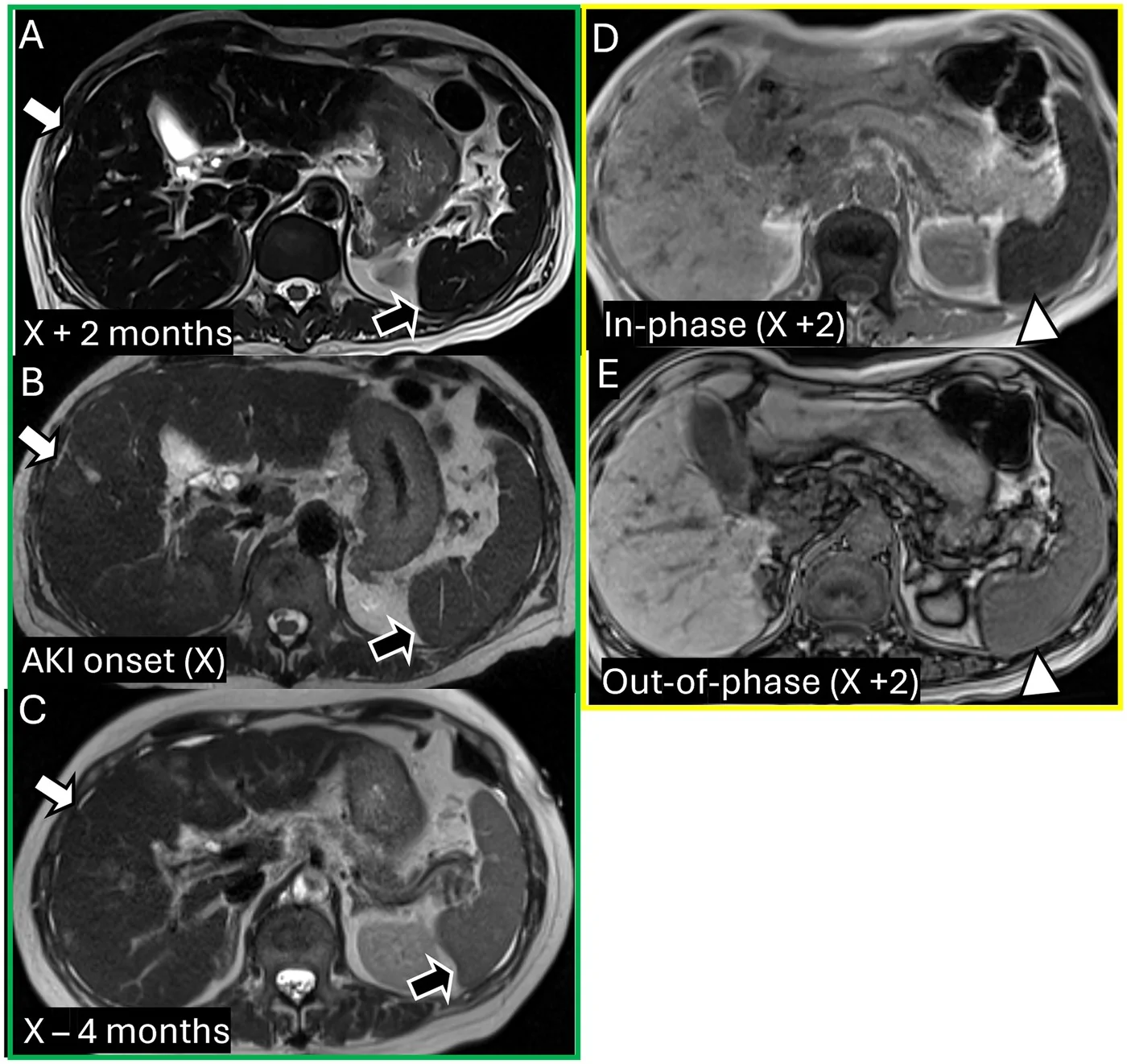
Evolution of systemic siderosis. Representative axial T2w images show progression of T2 hypointensity of the liver and spleen 2 months after AKI onset (A), compared to studies obtained at AKI onset (B) and 4 months prior (C). In-phase and out-of-phase imaging obtained at the time of the index study (X + 2, D and E, respectively) show loss of splenic signal intensity on in-phase imaging, compatible with iron deposition. These findings suggest secondary hemosiderosis. The iron deposition in the MPS (spleen and liver) manifested earlier in the disease course. When renal cortical iron deposition was detectable on imaging, the patient had begun to develop progressive renal injury.

The patient underwent a non-targeted renal parenchymal biopsy of the left kidney, which showed acute on chronic TMA. No immune complex deposition was identified. Based on pathology alone, the differential diagnosis for TMA includes aHUS, thrombotic thrombocytopenic purpura (TTP), and infection.

The radiologic differential diagnosis for renal cortical siderosis consists primarily of disorders resulting in intravascular hemolysis, including PNH, sickle cell anemia, and mechanical hemolysis related to mechanical valve replacement. Other causes of hemosiderosis (transfusion, inborn errors of iron metabolism) are less likely to manifest with renal siderosis. Given the pathological findings of TMA, the leading differential diagnoses were hemolytic disorders including hemolytic uremic syndrome, toxic thrombocytopenic purpura, and complement-mediated disorders including aHUS.

## Treatment

Clinically, the patient was diagnosed with aHUS given thrombocytopenia, elevated lactate dehydrogenase, and other findings of intravascular hemolysis with negative ADAMTS13 (excluding TTP). A trial of plasma exchange was unsuccessful as the patient’s cell counts dropped. The patient ultimately required hemodialysis and was initiated on Eculizumab, a terminal complement-pathway inhibitor, for aHUS. Unfortunately, the patient had altered mentation and was found to have acute and chronic parieto-occipital cortical infarcts, likely embolic or watershed. At the time of hospital discharge, she had returned to her baseline mental status, required hemodialysis, and had plans to continue Eculizumab infusions.

The long-term prognosis remains unclear. While lifelong complement blockade used to be the norm, newer data suggest this is not required for all patients.^3^ Additionally, case reports of this rare disease have shown return of renal function up to 18 months after initiation of complement blockade.^4^

## Discussion

Renal hemosiderosis is characterized by marked T2 hypointensity of the renal cortex and can be a sign of ongoing intravascular hemolysis.^5^ Depending on the etiology, T2 hypointensity of the medulla may also be seen.^6^ With intravascular hemolysis, other findings of secondary hemosiderosis may be present, including hepatic and splenic iron deposition. Relative loss of signal on in-phase compared to opposed-phase imaging may also be seen. The so-called “India ink” artifact caused by the loss of signal at fat-water interfaces is a critical problem-solving tool for abdominal imaging. In renal imaging, this artifact can be helpful in identifying subtle fat-rich angiomyolipomas.^7^ Elsewhere, this artifact can aid in characterization of lipomatous hypertrophy of the interatrial septum and focal pancreatic fat deposition.^7^

Renal cortical hemosiderosis can be seen in 3 general settings: intravascular hemolysis, vascular disease, or infection. The most common hemolytic cause of renal cortical hemosiderosis is intravascular hemolysis from mechanical heart valves. Early identification of renal cortical hemosiderosis allows for timely intervention, which may include adjustment of anticoagulation, transfusion, or valve revision. Therapy is aimed at treating the underlying cause of intravascular hemolysis, not the secondary iron deposition itself.

Renal cortical hemosiderosis is also classically seen in PNH, an acquired hematopoietic stem cell disorder in which dysregulation of the innate immune system leads to complement-mediated intravascular hemolysis. When left untreated, PNH can cause thrombosis, acute or chronic renal disease, pulmonary hypertension, and bone marrow failure. Additional causes include HUS/TTP, sickle cell disease, and thalassemia. Atypical HUS, unlike typical HUS, is a complement-mediated process like PNH with multiple triggers.^3^ Complement dysregulation leads to complement deposition on endothelial cells, particularly within the renal microvasculature. Subsequent aggregation of cellular debris creates a prothrombotic environment, leading to occlusive thrombi in the microvasculature. This causes renal injury and shearing of red blood cells (intravascular hemolysis),^8^ whereby processing of free hemoglobin leads to accumulation of iron in the monocyte phagocyte system (MPS) and renal parenchyma. While widespread, the pathology tends to affect the renal microvasculature most frequently.^8^ Atypical hemolytic uremic syndrome has several known triggers, including pregnancy, infection, or sepsis (which may have been the cause in this case), although it is idiopathic in up to 30% of cases.^9^ Notably, aHUS carries a poor prognosis and thus timely treatment, often comprising immunotherapy such as eculizumab or supportive treatment via transfusion and dialysis as in this case, is essential.

Vascular causes of renal hemosiderosis include renal vein thrombosis, arterial infarct, cortical necrosis, and rejection of a renal transplant—conditions in which early identification and treatment are critical as they can lead to progressive renal damage, loss of function, and systemic complications if left untreated. Infections are also an uncommon cause of renal cortical hemosiderosis. Hemorrhagic fever with renal syndrome (HFRS), caused by hantavirus, has been described in the literature. Most patients with HFRS make a full recovery with supportive care contingent on early and efficient fluid management. Antivirals such as ribavirin have demonstrated efficacy in clinical trials when administered in the early stages of the disease.^1^

Recognition of renal hemosiderosis can suggest an etiology of renal injury and may guide further diagnostic and therapeutic steps. For example, in an otherwise healthy patient with new-onset of hepatic vein thrombosis (Budd-Chiari syndrome) and renal cortical hemosiderosis, PNH can be considered. Identifying renal hemosiderosis via MRI can aid clinicians in efficiently narrowing their diagnostic focus to more quickly and effectively treat and manage the underlying cause.

## Learning points

- Renal cortical hemosiderosis is characterized by accumulation of iron in the renal cortex and is seen as marked T2 hypointensity of the renal cortex.
- Renal hemosiderosis can be seen in numerous settings, including hemolytic, vascular, and infectious causes. The most common cause is intravascular hemolysis related to mechanical heart valves.
- When combined with other clinical findings, identification of renal hemosiderosis via MRI can enable clinicians to more quickly diagnose and effectively address the underlying pathology.
- Atypical hemolytic uremic syndrome, a rare cause of renal failure, can cause renal cortical siderosis.
